# A randomized pilot trial evaluating safety and immunogenicity of recMAGE-A3 + AS15 immunotherapeutic administered by intramuscular versus intradermal/subcutaneous routes

**DOI:** 10.1186/2051-1426-2-S3-P60

**Published:** 2014-11-06

**Authors:** Craig L Slingluff, Gina R Petroni, Walter C Olson, Kimberly A Chianese-bullock, Kelly T Smith, Mark E Smolkin, Nadedja Galeassi, William Grosh, Geoffrey Weiss, Kristy Scott, Ana Hornillo

**Affiliations:** 1 University of Virginia, Charlottesville, USA; 2 Glaxo Smith Kline, Belgium

## Introduction

The recMAGE-A3 protein has been administered intramuscularly (IM) with immunostimulant AS15 as an experimental immunotherapeutic. AS15 contains 3-O-desacyl-4'-monophosphoryl lipid A (MPL), QS-21, CpG 7909 and liposome. This MAGE-A3/AS15 immunotherapeutic has not been studied for intradermal (ID) or subcutaneous (SC) use. A clinical trial (NCT01425749) was initiated to test the hypotheses that ID/SQ administration is safe and may induce CD4^+ ^and CD8^+ ^T cell responses to MAGE-A3.

## Patients and methods

Twenty-five eligible patients with resected stage IIB-IV MAGE-A3^+ ^melanoma were randomized to 2 arms, treated with MAGE-A3/AS15 Immunotherapeutic IM (Arm A, n = 13) or ID/SC (Arm B, n = 12). Adverse events (CTCAE 4) were recorded. Antibody (Ab) responses to MAGE-A3 protein were assessed by ELISA assay. T cell responses were assessed by flow cytometry after intracellular cytokine staining (ICS) for multifunctional CD4^+ ^and CD8^+ ^responses to overlapping MAGE-A3 peptides, assaying lymphocytes from peripheral blood (PBMC) and sentinel immunized node (SIN), after one *in vitro *stimulation.

## Results

In both arms, the recMAGE-A3/AS15 immunotherapeutic was well-tolerated, with only one grade 3 treatment-related adverse event (hyperglycemia, Arm B), and no grade 4 or 5 events. Grade 2 injection site reactions were observed in 10 patients in Arm A and 7 in Arm B (P > 0.3). Ab responses were detected in all patients, most with high titers persisting at least 6 months, without difference between arms. Preliminary T cell data are that multifunctional (IFNg and TNFα) CD4^+ ^T cell responses to MAGE-A3 were detected in 64% of patients (54% A; 75% B; Table 1). Multifunctional CD8^+ ^T cell responses were evident in 20% of patients (8% A, 33% B). CD4^+ ^responses were higher magnitude in SIN than in PBMC.

**Table 1:** Multifunctional (IFNg and TNFα) T cell responses to MAGE-A3

	% of CD4+ T cells			% of CD8+ T cells		
	(90% CI)			(90% CI)		
	SIN	PBMC	Either	SIN	PBMC	Either
Arm A	31%	31%	54%	0%	8%	8%
	(11, 58)	(11, 58)	(29, 78)	(0, 21)	(0, 32)	(0, 32)
Arm B	64%	50%	75%	18%	25%	33%
	(35, 86)	(25, 75)	(47, 93)	(3, 47)	(7, 53)	(12, 61)
Total	46%	40%	64%	8%	16%	20%
	(28, 64)	(24, 58)	(46, 80)	(2, 24)	(6, 33)	(8, 38)

## Conclusion

Safety profiles were comparable for ID/SC and IM administration of the MAGE-A3/AS15 immunotherapeutic, which induced high-titer Ab, multifunctional CD4^+ ^Th1 responses, and CD8^+ ^responses when administered by either route. Immune responses were more readily detected in the SIN than in PBMC. These pilot data support further investigation of ID/SC immunization with antigen plus AS15 to support Th1 CD4^+ ^responses and CD8^+ ^responses. Production of Th1 cytokines IFNg and TNFα suggests the induced CD4^+ ^responses may support CD8^+ ^T cells. Other forms of antigen (e.g.: long peptides) may further support induction of CD8^+ ^T cell responses in combination with AS15.

Funding source: GlaxoSmithKline Biologicals SA

